# Pop Up Satellite Tags Impair Swimming Performance and Energetics of the European Eel (*Anguilla anguilla*)

**DOI:** 10.1371/journal.pone.0020797

**Published:** 2011-06-08

**Authors:** Caroline Methling, Christian Tudorache, Peter V. Skov, John F. Steffensen

**Affiliations:** 1 Marine Biological Section, University of Copenhagen, Helsingør, Denmark; 2 Institute of Biology, Leiden University, Leiden, the Netherlands; 3 National Institute of Aquatic Resources, Section for Aquaculture, Technical University of Denmark, Hirtshals, Denmark; 4 DTU Aqua, National Institute of Aquatic Resources, Technical University of Denmark, Charlottenlund, Denmark; Institut Pluridisciplinaire Hubert Curien, France

## Abstract

Pop-up satellite archival tags (PSATs) have recently been applied in attempts to follow the oceanic spawning migration of the European eel. PSATs are quite large, and in all likelihood their hydraulic drag constitutes an additional cost during swimming, which remains to be quantified, as does the potential implication for successful migration. Silver eels (L_T_ = 598.6±29 mm SD, *N* = 9) were subjected to swimming trials in a Steffensen-type swim tunnel at increasing speeds of 0.3–0.9 body lengths s^−1^, first without and subsequently with, a scaled down PSAT dummy attached. The tag significantly increased oxygen consumption (MO_2_) during swimming and elevated minimum cost of transport (COT_min_) by 26%. Standard (SMR) and active metabolic rate (AMR) as well as metabolic scope remained unaffected, suggesting that the observed effects were caused by increased drag. Optimal swimming speed (*U*_opt_) was unchanged, whereas critical swimming speed (*U*_crit_) decreased significantly. Swimming with a PSAT altered swimming kinematics as verified by significant changes to tail beat frequency (*f*), body wave speed (*v*) and Strouhal number (St). The results demonstrate that energy expenditure, swimming performance and efficiency all are significantly affected in migrating eels with external tags.

## Introduction

The European eel (*Anguilla anguilla*) is common in waters of Western Europe. The spawning site of this species is believed to be the Sargasso Sea since this is where the smallest eel larvae have been found [1]–[3], but so far neither spawning adults nor eggs have been found in the Sargasso Sea to confirm this. European eel stocks have seen a strong decline since the 1980's and numbers are now believed to have declined by as much as 90 to 99% [4]. There are several hypotheses as to the causative mechanisms, including overfishing, pollution, and mass infections with the swim bladder parasite *Anguillicola crassus*. In addition, the nutritional status of individuals at the onset of migration may also play a role, in that many eels apparently do not have the minimum fat content required to fuel the journey [5]–[8]. Further insights into this part of the European eeĺs reproduction cycle, are very valuable for future management of the species both with regards to conservation and successful breeding programs in aquaculture. Several attempts have been made to follow eels during their spawning migration to gain information on the migration route and the direction. In many of these studies eels were tracked with acoustic transmitters, and individuals were only followed for a limited time, up to 156 hours [9]–[13]. During the past decades, externally attached pop-up satellite archival tags (PSATs) have frequently been used in tracking studies on a variety of large pelagic fish species [14]–[19]. PSATs make it possible to recover information on a multitude of parameters including temperature, depth and geo-location, and thus provide valuable information on swimming velocity, direction and depth that may help obtain a better understanding of the migratory behavior of European eel as this information could provide the key to their reproduction success and recent decline in population strength. PSATs have also been used on one of the largest eel species, the New Zealand longfin eel (*Anguilla dieffenbachii*) by Jellyman and Tsukamoto [20], who tracked 7.6–11.4 kg eels for 2–3 months, and recently, Aarestrup and co-workers [21] used PSATs to track migrating European eels for distances up to 1300 km. The time required to cover this distance was approximately 2 months and corresponded to a swimming speed between 0.06–0.3 body lengths per second. Despite energy spent on diel vertical migrations, the distance was shorter and the speed was slower than anticipated. Assuming a cruising speed of 0.8 to 1 body length per second, as suggested by Palstra and co-workers [22], a 1 meter long eel should be able to swim the 5000–6000 km from continental Europe to the Sargasso Sea in 3 to 4 months. Migrating European eel are much smaller than other species traditionally used in PSAT studies, and it is possible that the hydrodynamic resistance of the tag is a barrier to successful migration.

The objectives of the present study were to measure the energy expenditure at different swimming speeds, analyze the swimming capacity and the biomechanics of migratory silver eels equipped with a PSAT dummy, and to compare them with those of untagged eels.

## Materials and Methods

This study was carried out in accordance with the Danish Animal Experimentation Act and the protocol was approved by the Danish Animal Experimentation Board (licence number: 2004/561–894).

### Fish origin and husbandry

Eels were caught with traps in the vicinity of the Marine Biological Laboratory, University of Copenhagen, in October 2009. Fish were kept in a circular 3000 L tank, supplied with recirculating aerated seawater with a salinity of >32‰, at a constant temperature of 10°C. Eels were kept under these conditions to acclimatize for at least two months prior to experimentation. In accordance with the general observation of migrating silver eels, they did not feed, although offered a variety of food items. All eels were determined to be females in their migrating phase (Stage IV) [23].

### Setup

**Figure 1:**
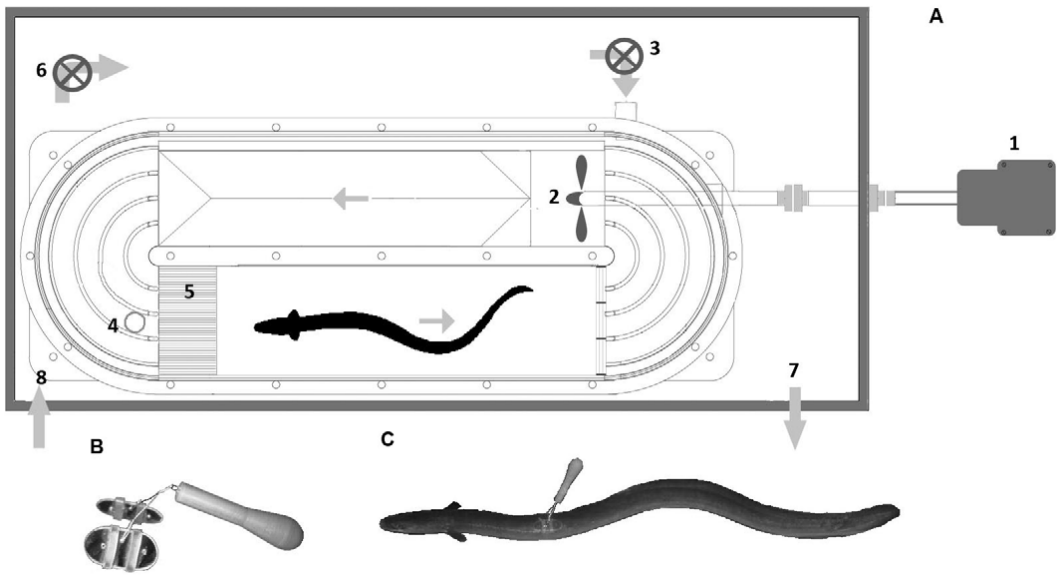
Schematic of swim tunnel and PSAT dummy. A. 1. Motor, 2. Propeller, 3. Flushpump (inlet), 4. Flush outlet, 5. Honeycomb, 6. Mixing pump, 7. Outlet from tank to water reservoir, 8. Inlet to tank from water reservoir. Arrows indicate water flow.B. PSAT dummy. C. PSAT dummy attached to eel. Refer to text for details.

Tests were performed in a 90 l Steffensen-type swim tunnel, downsized to 55 l by inserting a solid section, blocking the lower half of most of the tunnel leaving a 70*20*10 cm (l*w*d) swimming section (Fig. 1A). Turbulence was minimized by directing the flow through two sets of baffles and a 10 cm honeycomb. The swim tunnel was submerged in an outer tank, supplied with aerated water from a reservoir. The water in the outer tank was maintained at 10±0.1°C by continuously pumping it through a thermostat, a filter and an aquarium UV sterilizer. In addition, the water was kept well-mixed by a submerged Eheim-pump. Water velocity was controlled by a motor-driven propeller and motor controller (WEG, Germany) and the output voltage calibrated against a TAD flow meter (Höntzsch, Germany). Velocities were corrected for solid blocking effect according to Bell and Terhune [24]. A CCDTV video camera (TSR481, ELMO CO, LTD, Japan) was mounted above the swimming section illuminated with a single white LED allowing filming of the entire swimming section. The entire setup was shielded from daylight and other disturbances by black curtains. Video sequences were recorded by the PCTV USB2 software (Pinnacle systems Inc. CA, USA). Oxygen tension was continuously measured (1 Hz) with a Fibox 3 electrode by the Oxyview software (version 5.31, PreSense, Germany) and recorded by the AutoResp™ 1 software (version 1.6, Loligo systems). Intermittent flow through respirometry [25]–[27] was used to monitor the oxygen consumption at different swimming velocities. The swim tunnel was periodically flushed for 8 min with water from the outer tank, followed by a closed 2 min waiting period, to obtain steady state conditions, and a 20 min measuring period.

### Protocol

Three swimming trials were completed on 9 individuals, with the first serving as control without a tag for the subsequent two trials with a tag attached. The third trial was performed in order to investigate if swimming performance was affected by repeated trials. Further trials were not undertaken as preliminaries showed no difference between second and third trials. Before being introduced to the swim tunnel, eels were quickly (3–4 min) anaesthetized in a 40 mg L^−1^ benzocaine solution. Benzocaine is rapidly excreted across the gills with a half-life of ∼20 min [28]. Total length (L_T_ = 598.6±29 mm SD), mass (339.6±51.5 g SD), maximum height and width, were recorded to adjust for solid blocking and in addition to these, pectoral fin length, vertical and horizontal eye diameter were recorded to classify silver stage. Eels were left to acclimatize in the swim tunnel for 24 hrs at a velocity of 0.3 body lengths per second (BL s^−1^), corresponding to the lowest speed that incited swimming. After 24 hrs, the velocity was increased in increments of 0.1 Bl s^−1^ during the 2 min waiting period. Eels swam at each new speed for 20 min (the measurement period) and the speed was increased until they were unable to maintain swimming and keep off the rear grid. Eels were then removed from the swim tunnel, anaesthetized as above and the tag was attached. The tag was a scaled-down replica of a PSAT (X-tag Archival) (Microwave Telemetry, Inc. DC, USA). A scaled-down tag was chosen to match the size of the eels, as they were smaller than the migrating eels tagged with a PSAT in previous tracking studies [20], [21]. The PSAT dummy was manufactured from a cylindrical piece of PVC (16 mm in diameter, 60 mm long and mass in air 5.6 g), compared to 32 mm, 130 mm and 42 g of the original tag. The frontal cross-sectional area of the dummy tag was on average 24% of the cross-sectional area of the eel. As the original tag, the dummy tag was positively buoyant. The drag (g) of the tag was measured separately in a flow chamber with a force transducer, converted to mN and expressed as function of water velocity (cm s^−1^) by y = 0.013×^1.79^ (r^2^ = 0.99). The tag was attached to the eels by a stainless steel wire from the tag to two plastic attachment plates (30*15*2 mm) positioned on either side of the body, in order to evenly distribute the drag. The tag was positioned approximately ¼ of a body length from the snout, so that the lift from the tag would be approximately centred. The attachment plates were rounded and equipped with silicone pads to minimize stress to the skin, and attached to the eel by two parallel surgical steel wires (0.3 mm Ø) transversing the dorsal body musculature. The position and placement of the tag was in close similarity to the study by Aarestrup and co-workers [21]. The attachment of the tag was completed within 2 min, during which the gills were flushed with aerated water containing a weak dose (20 mg L^−1^) of anaesthetic. Eels were returned to the swim tunnel, where they were left to recover swimming at 0.3 Bl s^−1^. The swim trial was repeated as above 24 and 48 hrs after attachment of the tag.

### Calculations and statistics

Mass specific oxygen consumption (MO_2_) was derived from the decrease in oxygen partial pressure (pO_2_) during the 20 min measuring period according to: MO_2_ = V(*d*(pO_2_)/*d*t) αM^−1^, where V is volume of the swim tunnel, α is oxygen solubility and M is the wet weight. Oxygen consumption as a function of swimming speed (*U*) was fitted to the equation: MO_2_ = a*U* ^b^+SMR, with SMR being the standard metabolic rate at zero speed or at rest. The critical swimming speed (*U*_crit_) was calculated according to Beamish [29] as *U*_crit_ = *U*_f_+(t_f_t_i_^−1^Δ*U*) where *U*_f_ is the highest velocity maintained for an entire 20 min interval, Δ*U* is the velocity increment (5 cm s^−1^), t_f_ is the duration of the final (fatigue) velocity increment and t_i_ is the time interval (20 min; [30]). Active metabolic rate at the critical swimming speed (AMR_crit_), sustained for 20 min, was used to calculate the factorial metabolic scope (AMR_crit_ SMR^−1^). A polynomial equation (ax^2^+bx+c) was fitted to the relationship between fish swimming speed and oxygen consumption. The swimming speed with the lowest cost of transport (*U*_opt_) and the corresponding oxygen consumption (COT_min_) was calculated from the roots of the derivative as x = −b/2a and y = −(b^2^−4ac)/4a, respectively.

Video recordings from each swimming speed were analysed to calculate tail beat frequency (*f*), tail beat amplitude (*a*) and body wave velocity (*V*). Tail beat frequency was obtained by counting during a 20 second period at the beginning, middle and end of each swimming velocity. *a* was calculated using Vernier Logger Pro (v3.6., Vernier Software & Technology, USA). Frames where the tail was in the outermost position were chosen and position of the tail tip recorded. The amplitude was calculated as the difference between the two outermost positions of the tail tip during one tail beat. This was repeated ten times for each of the three periods, and used to calculate an average for each swimming speed. Body wave velocity (*V*) was calculated as the distance travelled by a wave crest over time from the digitized video sequences using Vernier Logger Pro. The Strouhal number (St) was calculated according to the formula St = *af*/*U*. The Strouhal number is dimensionless and has been shown to be strongly correlated to force production and efficiency of flapping foils [31] and the propulsive efficiency of swimming fish [32], [33].

Data of tagged and untagged eels were compared at each swimming speed using repeated measurements ANOVA followed by a Holm-Sidak multi comparison procedure (SigmaPlot v. 11, Systat systems inc. USA) when significant effects were found. Significance value was p<0.05.

## Results

### Respirometry

**Figure 2:**
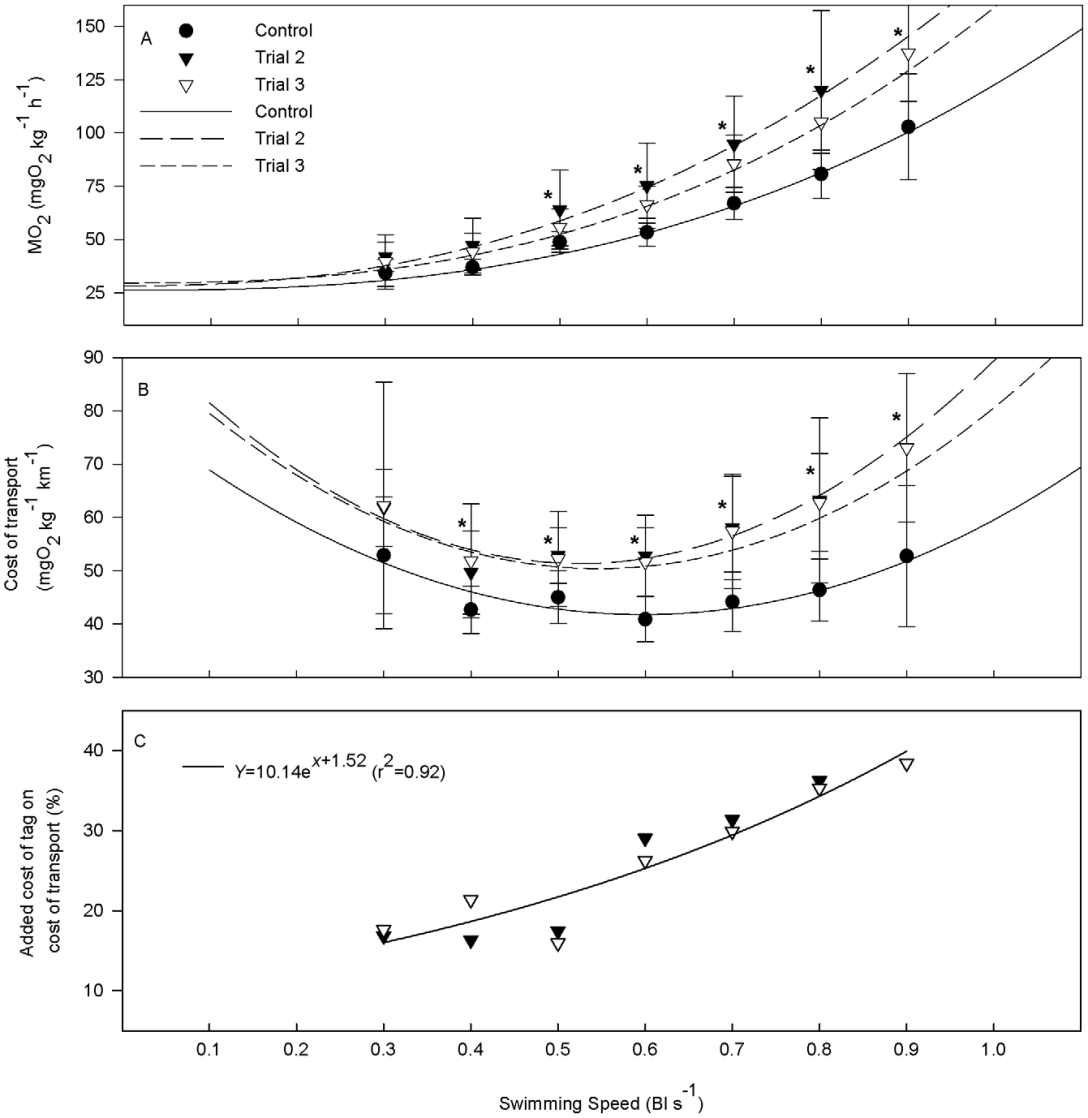
Swimming energetics in (*A. anguilla*) with a PSAT dummy. A. Oxygen consumption (MO_2_, mgO_2_ kg^−1^ h^−1^) and B. Cost of transport (COT, mgO_2_ kg^−1^ km^−1^) as a function of swimming speed (*U*, Bl s^−1^) swimming with and without a PSAT dummy. Swim trials without (control) and with a tag (trials 2, 3) were performed on the same individual (*N* = 9). Lines are regression lines (refer to Table 1 for regression values). An *asterisk* denotes significant difference between control and tagged condition (Repeated measures ANOVA, p<0.05). Data are presented as mean±SD. C. Additional cost of tag on COT as a function of swimming speed (*U*, Bl s^−1^). Line is regression line of the average value of trials 2 and 3.

Oxygen consumption during swimming was significantly higher for tagged compared to non-tagged eels (Trial 1 v. Trials 2, 3) at speeds above 0.4 Bl s^−1^ (Fig. 2A). There was no significant difference between Trial 2 and Trial 3. There were no significant differences in standard metabolic rate (SMR), active metabolic rate (AMR_crit_) or metabolic scope between trials. Cost of transport was significantly higher in tagged versus untagged eels at all but the lowest swimming speed (0.3 BL s^−1^), again with no difference between tagged trials (Fig. 2B). The critical swimming velocity *U*_crit_ was significantly lower with a tag attached compared to control, but there was no difference between tagged trials (Table 1). Optimal swimming speed (*U*_opt_) was not affected by tagging, but minimum cost of transport (COT_min_) was significantly higher when swimming with a tag (Table 1). The additional cost of swimming with the tag, on cost of transport, increased with swimming speed according to the formula (average of trials 2 and 3) y = 10.14e^1.52x^ (r^2^ = 0.92) and was estimated to be 26% higher at the optimal swimming speed *U*_opt_ of 0.6 Bl s^−1^(Fig. 2C).

**Table 1:** Regression values and swimming energetic parameters.

Parameter	Control	Trial 2	Trial 3
a	96.62±38.64^a^	148.79±56.28^a^	129.58±25.17^a^
b	2.51±1.24^a^	2.29±0.67^a^	2.51±0.98^a^
SMR (mgO_2_ kg^−1^ h^−1^)	26.28±11.24^a^	28.36±7.42^a^	29.71±13.70^a^
	(r^2^ = 0.993)	(r^2^ = 0.995)	(r^2^ = 0.985)
AMR (mgO_2_ kg^−1^ h^−1^)	106.83±29.39^a^	118.09±35.99^a^	112.15±34.91^a^
Scope	5.15±3.77^a^	4.46±2.01^a^	4.04±1.11^a^
*U*_opt_ (Bl s^−1^)	0.60±0.12^a^	0.54±0.06^a^	0.52±0.07^a^
COT_min_ (mgO_2_ kg^−1^ km^−1^)	40.70±2.27^a^	50.87±5.35^b^	49.58±7.47^b^
*U*_crit_ (Bl s^−1^)	0.90±0.14^a^	0.73±0.13^b^	0.80±0.13^b^

Oxygen consumption (MO_2_, mgO_2_ kg^−1^ h^−1^) was expressed as a function of swimming speed (*U*, Body lengths s^−1^) with the formula *f* = ab*U* ^b^+SMR. Swim trials without (control) and with a tag (trials 2, 3) were performed on the same individual. Abbreviations: SMR, standard metabolic rate; AMR, active metabolic rate; *U*_opt_, optimal swimming speed; COT_min_, minimum cost of transport; *U*_crit_, critical swimming speed. Values are mean±SD. Different superscripts indicate significant differences per row (repeated measurements ANOVA, p<0.05, *N* = 9).

### Kinematics

**Figure 3:**
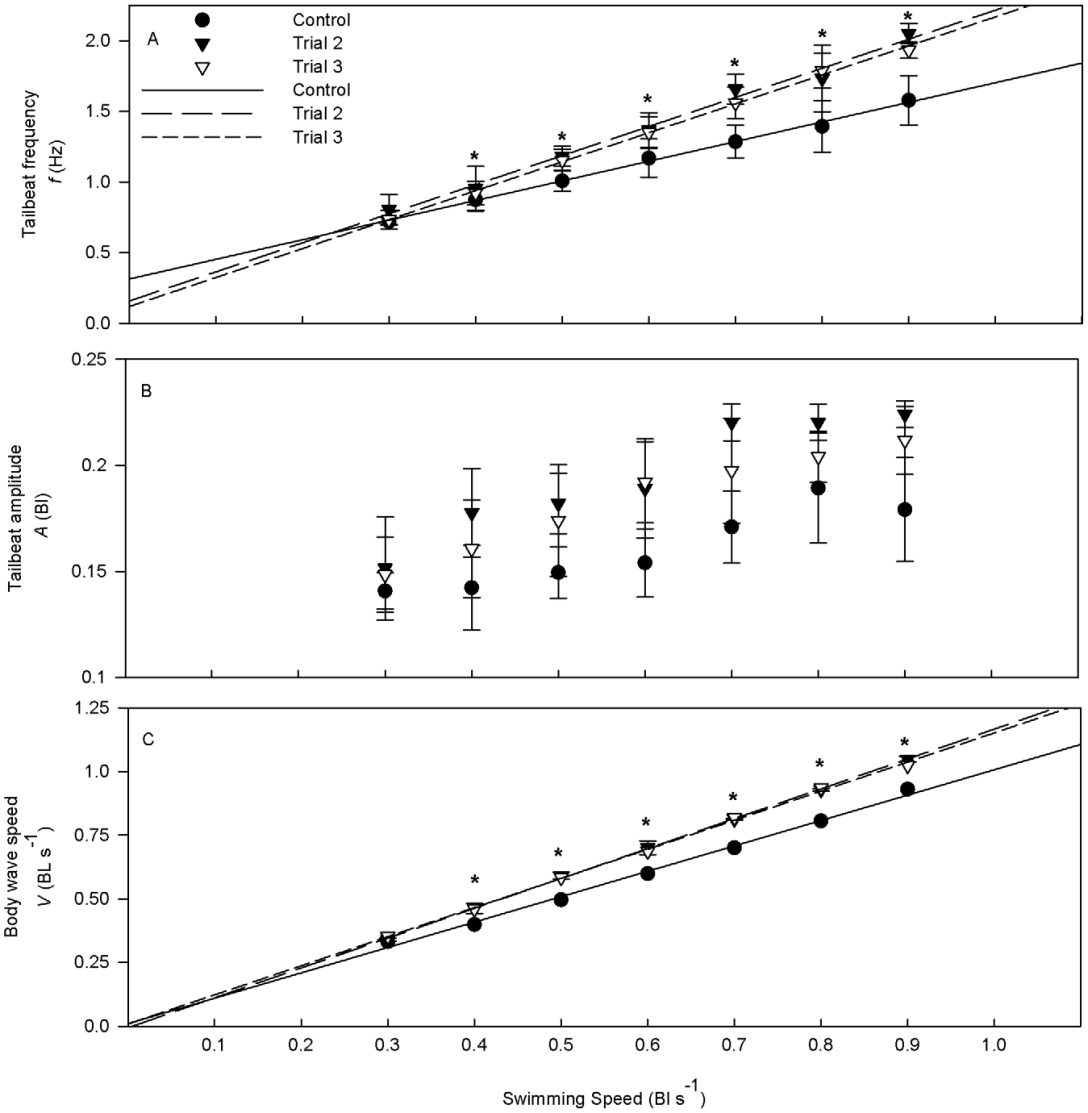
Swimming kinematics in (*A. anguilla*) with a PSAT dummy. A. Tail beat frequency (Hz) B. Tail beat amplitude (body lengths) and C. Body wave speed (body lengths) as a function of swimming speed (*U*, body lengths s^−1^) swimming with and without a PSAT dummy. Swim trials without (control) and with a tag (trials 2, 3) were performed on the same individual (*N* = 9). Lines are regression lines (refer to Table 2 for regression values). An *asterisk* denotes significant difference between control and tagged condition (Repeated measures ANOVA, p<0.05). Data are presented as mean±SD.

Tail beat frequency *f,* plotted against swimming speed (*U*) revealed a linear relationship, *f* = a+b*U*, with a being the intercept and b being the slope of the curve (Table 2). At swimming speeds greater than 0.4 Bl s^−1^ *f* was significantly higher in tagged eels compared to non-tagged eels at the same swimming speed (Fig. 3A). Tail beat amplitude (*a*) was not affected by tagging and remained constant across all swimming speeds at 10.57±1.38 cm (Fig. 3B). The width of the swim tunnel was 20 cm and thus the eels were allowed their full range of motion without any obstructions at all speeds. Body wave velocity (*V*) was positively correlated with *U* and could be described by *V* = a+b*U* (Table 2). At swimming speeds above 0.3 Bl s^−1^ *V* was significantly higher in tagged eels compared to non-tagged eels (Fig. 3C). The Strouhal number (St) was significantly lower in the control trial compared to the two tagged trials, with no difference between tagged trials.

**Table 2:** Regression values and swimming kinematics.

Parameter		Control	Trial 2	Trial 3
Tail beat frequency, *f*	a (slope)	1.41±0.21^a^	2.17±0.17^b^	2.11±0.17^b^
	b (intercept)	0.31±0.07^a^	0.15±0.08^b^	0.10±0.05^b^
		(r^2^ = 1.00)	(r^2^ = 0.99)	(r^2^ = 1.00)
Wave speed, *V*	a (slope)	1.01±0.02^a^	1.19±0.03^b^	1.15±0.02^a^
	b (intercept)	0.01±0.01^a^	0.02±0.01^a^	0.01±0.01^a^
		(r^2^ = 1.00)	(r^2^ = 1.00)	(r^2^ = 1.00)
Strouhal (St)		0.31±0.02^a^	0.43±0.05^b^	0.42±0.05^b^

Tail-beat frequency (*f*) and body wave speed (*V*) was expressed as a function of swimming speed (*U*, Body lengths s^−1^) with the formula *f* = a+b*U*. Swim trials without (control) and with a tag (trials 2, 3) were performed on the same individual. Values are mean±SD. Different superscripts indicate significant differences per row (repeated measurements ANOVA, p<0.05, *N* = 9).

## Discussion

This study demonstrates that attaching a PSAT dummy to European eels, results in an increased oxygen uptake during swimming, an increased cost of transport, and a decreased swimming efficiency and performance.

No changes to standard metabolic rate, active metabolic rate or metabolic scope were associated with fitting eels with a PSAT dummy. This implies that there was no increased energy expenditure during rest, for example from maintaining buoyancy, and that aerobic capacity was uncompromised. The standard metabolic rate was similar and within range of what is reported in the literature *for A. anguilla* [34] and *A. rostrata* [35], and for resting *A. anguilla* of similar size at the same temperature (unpubl. obs.). The increased oxygen uptake during swimming in the tagged trials was likely to be a result of the additional drag of the transmitter as SMR was not significantly increased. This has previously been suggested with regards to other externally attached transmitters [36]–[38] and recently, a similar observation of increased oxygen uptake during swimming was reported in a study on Atlantic cod (*Gadus morhua*) with an externally attached acoustic dummy transmitter [39]. They found no difference in standard or active metabolic rates, but a decrease in the optimal and critical swimming speed. In the present study, the maximum metabolic rate was reached at a lower swimming speed in tagged eel, which corroborates an additional metabolic cost of swimming with the tag. The values for AMR and *U*_crit_ were slightly lower than previously reported for silver eels of similar size [40]; Palstra and co workers reported an AMR of ∼120 mgO_2_ kg^−1^ h^−1^ and *U*_crit_ of ∼1.1Bl s^−1^, and Quintella and co workers [41] a *U*_crit_ of ∼1.2 Bl s^−1^. However, those studies were performed at 18°C compared to 10°C in the present study, which may explain the discrepancy, as it is generally observed that performance depends on ambient temperature [42], [43], and is at its maximum at the preferred temperature, that has been reported to be approximately 18°C in the closely related American eel (*A. rostrata*) [44]. In the present experiment a temperature of 10°C was chosen since this represents what has been demonstrated to be the average temperature for a considerable part of the journey to the Sargasso Sea [21]. Additionally, differences in set up size can account for the differences in results, as it has been shown previously [45]–[47]. Eels migrating in the wild might swim at very different optimal and cruising speeds, than found in any of these studies, due to the experimental set up. However, as ours and the other studies mentioned are comparative studies, the findings are conclusive. The optimal swimming speed did not decrease significantly when the eels were tagged. Swimming at a reduced *U*_opt_ would minimize the additional cost of swimming with a tag, but assuming that eel migrate at their optimal swimming speed, would also prolong the journey for a tagged individual, who then perhaps would not reach the spawning area in due time. Even though *U*_opt_ did not change it has to be kept in mind that the energy consumption was 26% higher for the tagged animals swimming at *U*_opt_. Recently, Quintella and co workers [41] reported that male and female silver eels had the same absolute critical swimming speed, measured in meters per second, despite a 37% difference in length. The authors proposed that this, by way of natural selection, was to favour the synchronized arrival at the spawning grounds. That study did not consider swimming energetics, but clearly a comparison of the optimal swimming speed and bioenergetics of male and female silver eels would be of interest in future studies. The minimum cost of transport reported herein was very similar to recently reported values for silver eels (*A. anguilla*) of comparable sizes i.e. Palstra and co workers [40] reported a value of 40 mgO_2_ kg^−1^ km^−1^ in eels not infected with the swim-bladder nematodes at 18°C. The approximately 26% increase in swimming cost (at the optimal swimming speed) that was associated with the tag found in the present study translates into an increased use of body fats. The average fat content of an eel is about 20%, ranging from 10–28% [48], so an average 1 kg eel would then have 200 grams of fat available. Swimming without and with a tag to the Sargasso Sea would cost 3.43 MJ and 4.29 MJ respectively if swimming at *U*_opt_. Assuming this is solely powered by body fat (37 kJ gram^−1^) an eel would use 93 or 115 grams of fat to complete the journey, which is 57.5% of its fat reserves. Completing the journey with a tag would then require a minimum body fat content of 12%, leaving no reserves left for gonad development and egg production. This leads to the assumption that the fat content of eels may be a critical aspect involved in their capacity to complete their migrating journey. The increased added cost of transport due to the tag can only be minimized by migrating at a lower speed (Fig. 2C). It is assumed that the duration of the European eeĺs spawning migration is approximately 4–6 months, so if swimming speed is reduced to avoid the increased cost of a tag the result will be a much shorter distance travelled in the mean time. Aarestrup and co workers [21] reported that European eel with a PSAT attached, had an average horizontal migration speed of 13.8 km day^−1^ for (∼0.16 Bl s^−1^), which is much lower than the speed required to complete the journey within the assumed time (about 0.5 Bl s^−1^) [49]. In addition, the furthest distance travelled was 1.300 km, compared to the necessary 6000 km to reach the hypothetical spawning grounds. When taking the observed daily vertical movement of 800 meters additional to the horizontal migration into account [21], however, the actual swimming speed would be somewhat higher, but still far from the required cruising speed. Nevertheless, the drag of this type of PSAT is possibly too high even for large specimens, which was also partly concluded by Jellyman and Tsukamoto [20], who tagged the much larger New Zealand longfin eel (*Anguilla dieffenbachii*) with PSATs. The dummy used in the present study had a transmitter:fish mass ratio of ∼1.7% in air, which is just under the 2% rule of thumb generally applied in tagging studies [38], [50], [51], and this suggests that drag is the more important factor to consider when using this type of tag on this size of fish. Although the drag of the dummy tag was only approximately 6 mN at the optimal swimming speed, it still significantly affected both the swimming performance, and the energy expenditure. It shows that external tags even of relatively low drag and buoyancy can have adverse effects on the animals carrying them. How the drag of the dummy tag relates to the drag of the eel is not known as it was not attempted to measure the drag of the eel. Unfortunately it is not straight forward to accurately measure the drag of a swimming eel due to the anguilliform swimming mode.

Tail beat frequency and body wave speed were both positively correlated with swimming speed, and both increased when eels were swimming with the tag. This can explain the increased MO_2_ and COT that was associated with the tag, because the muscles had to contract at a higher frequency to keep the same swimming speed. Tail beat frequency as a function of *U* predicted tail beats of 0.1–0.3 Hz at zero swimming speed, and hence was not the best predictor of swimming speed in the present study. Body wave speed, on the other hand, was a better predictor of *U* since the intercept was very close to zero, due to its close relationship to the product of tail beat frequency and amplitude. Similar observations were reported by Tytell [52], in the closely related American eel (*A. rostrata*).

The Strouhal number is used as a measure of the biomechanical swimming efficiency, with an optimal value of 0.3 signifying high efficiency of propulsion [31], [32]. According to this, swimming without the tag was very efficient and similar to what was observed in *A. rostrata* [52]. Moreover, amplitude did not change with swimming speed, which was also the case with *A. rostrata* [52]. The Strouhal number was higher in tagged trials, indicating that the tag made swimming less efficient, also reflected in the increased oxygen uptake and cost of transport. With both energetic and kinematic variables, the effect of the tag was minimal (not significant) at the lowest swimming speeds (0.3–0.4 Bl s^−1^), suggesting that the drag of the tag at those speeds (∼2–3.5 mN) was negligible.

It has been shown in the past that externally attached tags with even relatively low drag and buoyancy impair the swimming capacity of many diving species. Examples are Adélie penguins [53], [54] and cownose ray [55]. Size and form of the PSATs currently available are very much dependent on a) the size of the battery used and b) the size and form of the swimming device ensuring enough positive buoyancy to rise to surface and send the data to the satellite. The actual electronic devises responsible for measurement and storage of the parameters detected only account for a fraction of the weight and the drag responsible for the impairment of swimming. Additionally, an internal position of the tag would only impair the free rising of the devise to the surface for transmitting the data to the satellite, as seawater blocks radio waves. A perfect devise for following migrating eels would have a minimal drag and size and be neutrally buoyant as not to impair the daily vertical movement of migrating eels, observed by Aarestrup et al. [21].

In summary, the present study shows that the currently available PSATs are not suitable to be fitted to migrating eels, due to their increase in drag and the associated swimming costs by significantly affecting energy expenditure, swimming performance and efficiency in spite of the assumedly small transmitter:fish ratio and the low drag. We suggest that further studies should be made with a range of different sized tags to determine the optimal tag:eel ratio that makes it possible to track eels even further and longer.
